# Integrated control of a nanoindenter and X-ray nanodiffraction for automated *in situ* nanomechanical studies

**DOI:** 10.1107/S1600577526003723

**Published:** 2026-05-06

**Authors:** Anton Davydok, Otto Carlos Lippmann, Kritika Singh, Satishkumar Kulkarni, Christina Krywka

**Affiliations:** a Institute of Materials Physics Helmholtz-Zentrum Hereon Outstation at DESY, Notkestr. 85 22607Hamburg Germany; b Centre for X-ray and Nano Science CXNS Deutsches Elektronen-Synchrotron (DESY) 22607Hamburg Germany; POSTECH, Republic of Korea

**Keywords:** *in situ* nanomechanical testing, scanning X-ray nanodiffraction, beamline control software

## Abstract

X-ray diffraction (XRD) combined with *in situ* mechanical testing is a powerful, non-destructive technique that provides valuable information on structural evolution and defect formation during deformation. Unlike many other characterization methods, XRD does not impose constraints on sample dimensions, does not require a vacuum environment, and can be applied to a wide range of materials with periodic structures. However, such experiments present significant challenges due to the need for precise synchronization between independently controlled systems: one managing mechanical loading and the other handling X-ray measurements. In this work, we report on the successful software-level integration of a nanoindenter control system directly into the beamline control system. This integration enables full control of both mechanical and diffraction measurements from a single user interface, allowing real-time synchronization and automation of complex *in situ* experiments. We demonstrate the capabilities of this setup through uniaxial compression tests on α-Ti micropillars. Mechanical data from the nanoindenter and XRD patterns were collected simultaneously in an automated mode. Raster scans were performed on the micropillar in its pristine state, at two intermediate deformation stages, and post-mortem. This approach enabled detailed analysis of mechanical behavior and structural evolution under load, illustrating the effectiveness of the integrated system for advanced *in situ* studies.

## Abstract 2

This work presents a software-level integration of nanoindenter control directly into a beamline control system, enabling fully synchronized and automated *in situ* experiments through unified, real-time coordination of mechanical loading and X-ray nanodiffraction measurements.

## 1. Introduction

Understanding material behavior under mechanical load at small length scales is crucial for advancing structural and functional materials (Gianola *et al.*, 2023 ▸). *In situ* mechanical testing techniques offer a unique perspective by enabling simultaneous monitoring of mechanical response and microstructural evolution. This real-time insight is particularly valuable for studying processes such as plastic deformation, twinning mechanisms, phase transitions, and defect dynamics (Guo *et al.*, 2025 ▸; Wang *et al.*, 2018 ▸; Ball *et al.*, 2024 ▸; Kiener *et al.*, 2023 ▸).

Among various analytical techniques, X-ray diffraction (XRD) stands out as a particularly powerful method for material characterization. One of the most compelling advantages of XRD techniques lies in their non-invasive nature. Unlike electron microscopy methods, which require vacuum conditions and are often limited by sample size, geometry, and preparation constraints, XRD can be performed under ambient environments, often without complex sample conditioning (Nautiyal *et al.*, 2020 ▸). This characteristic significantly broadens the range of possible experimental configurations and makes XRD especially suitable for *in situ* mechanical testing, where observing the real-time response of materials to stress or strain is vital (Li *et al.*, 2024 ▸). Furthermore, XRD techniques can be used across a diverse set of materials, including metals, ceramics, polymers, and composites, underlining their versatility and broad applicability (Vullum *et al.*, 2006 ▸; Wang *et al.*, 2024 ▸; Jiang *et al.*, 2025 ▸).

Despite the clear advantage of XRD for *in situ* studies, fully integrated systems that combine micromechanical testing and synchrotron X-ray diffraction in real time are rarely found at beamlines. Several landmark studies have highlighted the potential of such integrated systems, albeit with limitations. One example is the use of Bragg coherent diffraction imaging (BCDI) in conjunction with a self-developed atomic force microscopy-based nanoindenter, which allowed imaging of strain fields at the nanoscale during nanoindentation (Cornelius *et al.*, 2012 ▸). While this setup demonstrated the concept of combining mechanical loading with high-resolution diffraction, it notably lacked real-time force feedback limiting the precision of mechanical control during deformation.

Also, *in situ* loading was successfully coupled with microLaue diffraction, enabling orientation mapping during mechanical testing (Kirchlechner *et al.*, 2011 ▸). These studies illuminated the ability of Laue-based techniques to resolve local lattice rotations and elastic strains in single crystal nanowire. However, such configurations often involve substantial manual alignment, making them difficult to integrate fully into synchrotron beamline environments.

More recently, a nanoindenter specifically designed for use at a synchrotron beamline has been reported by our group. A custom-built nanoindenter was used for XRD with *in situ* uniaxial compression tests, yielding insights into lattice evolution under load (Todt *et al.*, 2021 ▸; Giuntini *et al.*, 2021 ▸). Nevertheless, this setup still suffered from a lack of automatization and beamline integration, requiring the user to operate the mechanical testing system and X-ray data acquisition in separate, manually coordinated environments.

In a first attempt to overcome these shortcomings, we have implemented a commercial nanoindenter system (Oxford Instruments, FemtoTools NMT04) at our beamline. This setup has enabled automated fatigue testing of micropillars and microbeams, offering a commercial platform that achieves high force and displacement sensitivity (Zauner *et al.*, 2022 ▸; Janknecht *et al.*, 2025 ▸).

Emerging work using coherent diffraction imaging techniques under mechanical load has further demonstrated the rich potential of fully synchronized XRD and micromechanical measurements. For example, recent studies on β-phase titanium micropillars have showcased the sensitivity of XRD-based imaging to localized strain distributions during uniaxial compression (Ribart *et al.*, 2023 ▸). Similarly, the application of FemtoTools systems for *in situ* studies of fatigue and failure mechanisms has expanded the breadth of possible experiments (Jakob *et al.*, 2025 ▸).

Related work at other synchrotron facilities has also demonstrated integration of commercial nanoindenters into beamline control systems; for example, Lotze *et al.* (2024 ▸), who implemented a TANGO device server for remote operation of the Alemnis nanoindenter. While this approach enabled parallel control and remote access, the mechanical testing system remained largely decoupled from the beamline control necessitating significant user intervention to synchronize loading protocols with X-ray measurements.

In this work, we present a significant advancement in the realization of *in situ* nanomechanical experiments at synchrotron beamlines through the direct integration of a commercial nanoindenter control system into the beamline control software via the TANGO controls framework (Chaize *et al.*, 2001 ▸). Unlike previously reported nanoindentation–nanoXRD implementations, where mechanical testing and X-ray acquisition systems were typically operated in parallel but within largely decoupled control environments, the primary novelty of our approach lies in the complete software-level embedding of the nanoindenter into the beamline infrastructure. This is achieved through a custom-developed TANGO device server that incorporates the nanoindenter as a native device within the Sardana control framework, enabling unified operation through the user interface. As a result, mechanical loading and X-ray nanodiffraction measurements can be executed in a fully synchronized and automated manner from a single control environment, with real-time bidirectional communication and software-based trigger coordination. Complex experimental sequences such as chained raster-scanned nanoXRD mappings combined with multi-step loading and hold protocols can thus be performed without manual intervention. We demonstrate the capabilities of this integrated system through *in situ* uniaxial compression experiments on α-Ti micropillars, where synchronized acquisition of mechanical data and spatially resolved diffraction patterns allows direct correlation between applied load and microstructural evolution. This unified architecture substantially reduces experimental complexity, enhances reproducibility, and enables high-throughput, user-friendly *in situ* nanomechanical studies within the standard beamline workflow.

## 2. Experimental setup

The system was installed at the Nanofocus Endstation of the P03 beamline at the PETRA III synchrotron, DESY (Hamburg, Germany) (Krywka *et al.*, 2013 ▸). The objective of this endstation is to deliver a nanofocused beam with a long focal distance, enabling enhanced space for extended sample environments and facilitating high spatial resolution scanning X-ray nanodiffraction measurements with *in situ* and operando modes. During the experiment reported herein, XRD data were recorded from a 30 µm high α-Ti alloy micropillar while it was being compressed by a nanoindenter. The photon energy was set to 19.7 keV using a double-crystal monochromator and the X-ray beam was focused using a Kirk­patrick–Baez (KB) mirror system to a size of 1 µm × 1.1 µm (H × V). The proof-of-principle experiment presented here was intentionally conducted using the mentioned beam size rather than a sub-micrometre focus. This choice ensured reliable diffraction statistics while validating the synchronized control architecture. A beam size of 1 µm enables efficient data acquisition while providing sufficient spatial resolution for materials with comparatively large microstructural length scales, such as coarse-grained metals like in the presented case or biological materials. Sub-micrometer nanodiffraction experiments have also been successfully performed and will be reported elsewhere. The FemtoTools nanoindenter was mounted on a high-load piezo-driven stage from SmarAct, equipped with three sets of independent linear motors providing a step size resolution of 100 nm and a travel range of 14 mm in three orthogonal directions. Initial sample alignment was enabled by a side-view optical microscope, which is critical for positioning and tilting the sample surface relative to the nanoindenter tip. The final, precise alignment of the micropillar relative to the X-ray beam was done by using an Amtex X123 X-ray fluorescence detector. The diffraction signal was recorded using a Dectris Eiger 9M detector positioned at a distance of 201.1 mm from the sample, calibrated using an LaB_6_ diffraction standard.

**Figure 1:**
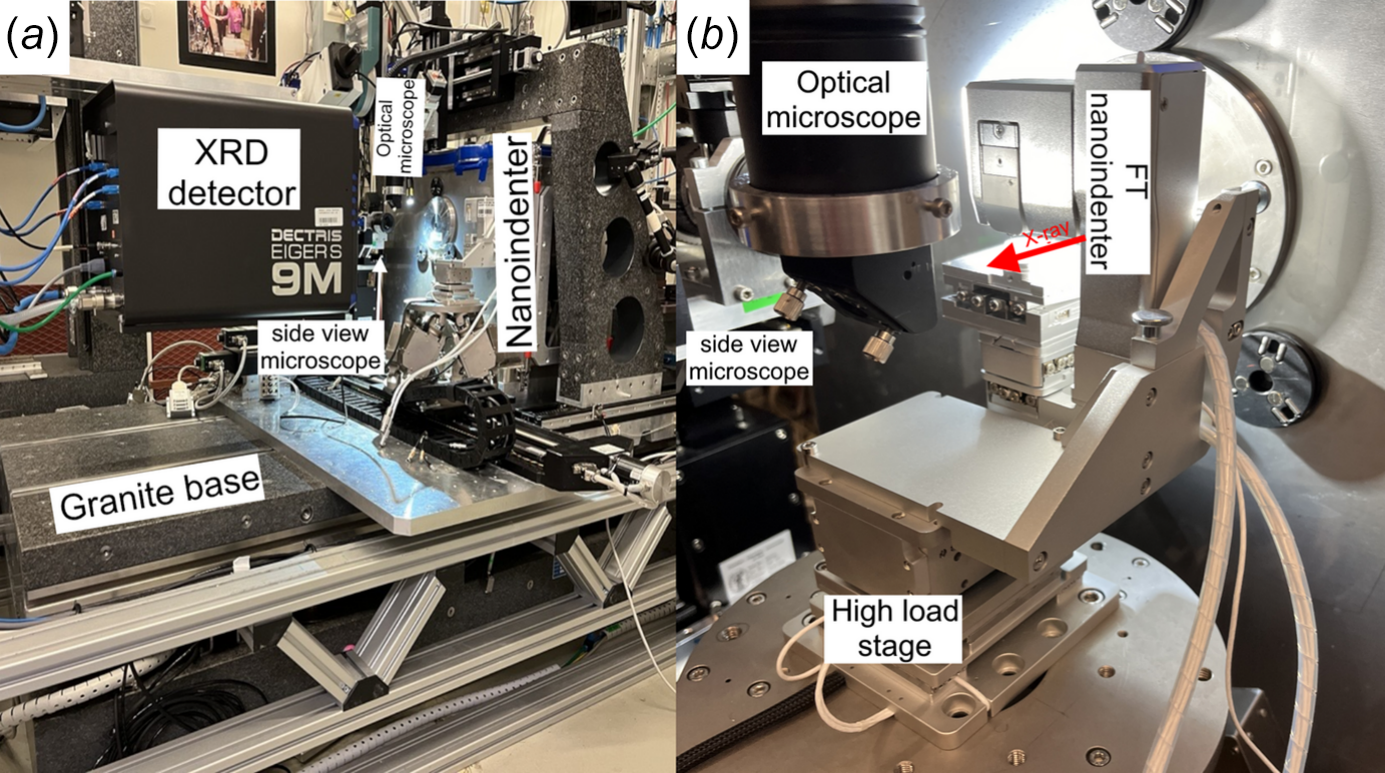
(*a*) Nanofocus endstation of P03 beamline with installed FemtoTools nanoindenter. (*b*) Photograph of the nanoindenter at the measurement position.

Fig. 1 ▸ shows the experimental setup at the Nanofocus Endstation of the P03 beamline, which is specifically designed to meet the high mechanical stability requirements of nanofocusing X-ray optics. This stability is achieved through the use of granite bases: a large granite block serves as the main foundation of the endstation [Fig. 1 ▸(*a*)], effectively minimizing vibrations and thermal drift which are important for nanomechanical experiments as well. The FemtoTools FT-NMT04 nanoindenter mounted at the beamline is shown in Fig. 1 ▸(*b*).

### 2.1. Sample preparation

The micropillar was fabricated at DESY NanoLab (Stierle *et al.*, 2016 ▸) from a bulk α-titanium alloy using dual-beam focused ion beam (FIB) milling with a gallium ion source (FEI Scios). An ion energy of 10 kV and 9.4 nA was employed, utilizing a coarse milling protocol to shape the microstructure. The resulting micropillar measured 10 µm in diameter and 30 µm in height, yielding an aspect ratio of 3, which is typical for uniaxial compression experiments (Fei *et al.*, 2012 ▸)

### 2.2. Software implementation

**Figure 2:**
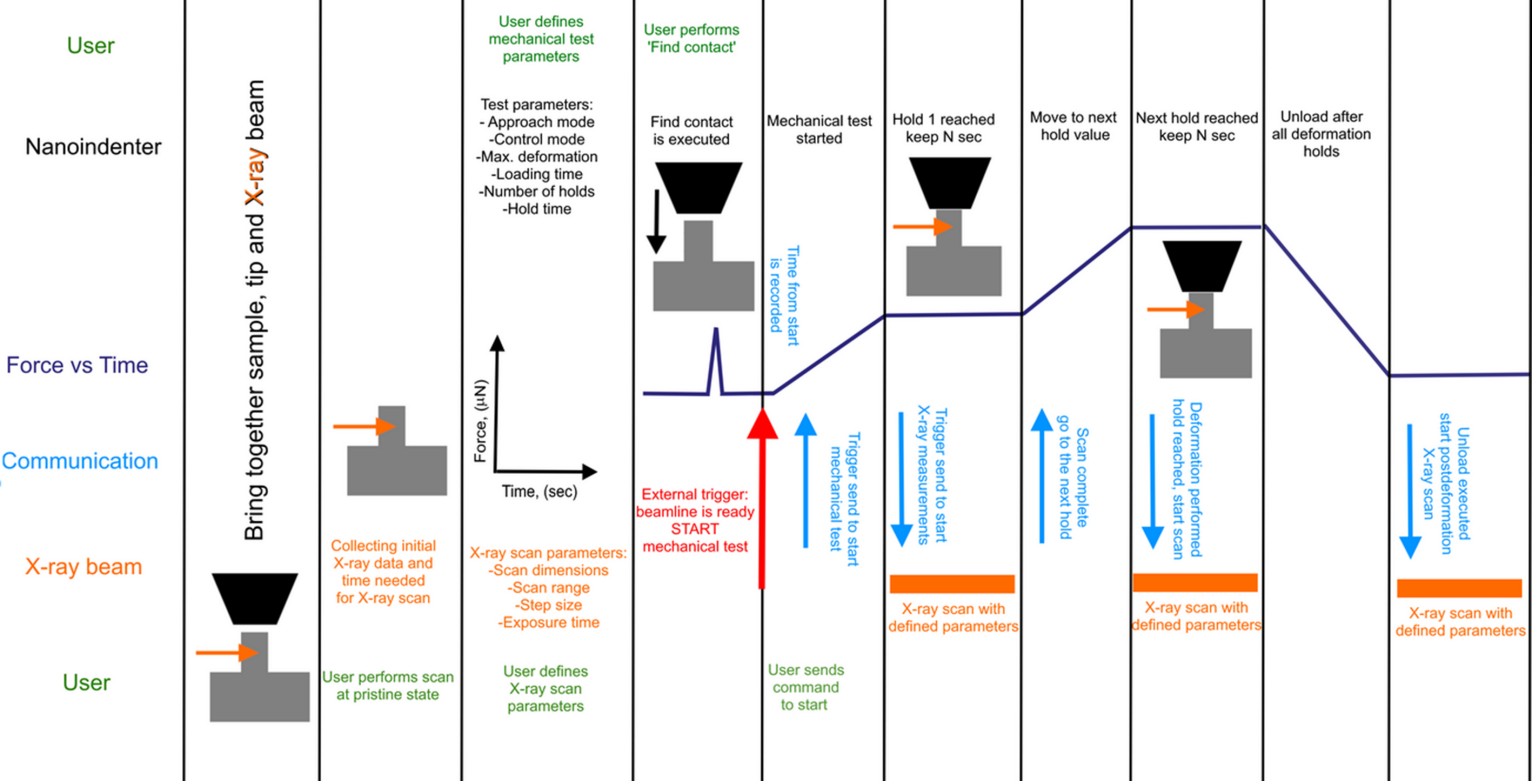
Schematic representation of the communication protocol between the FemtoTools *Remote Control* software and the TANGO device server during a micromechanical test.

**Figure 3:**
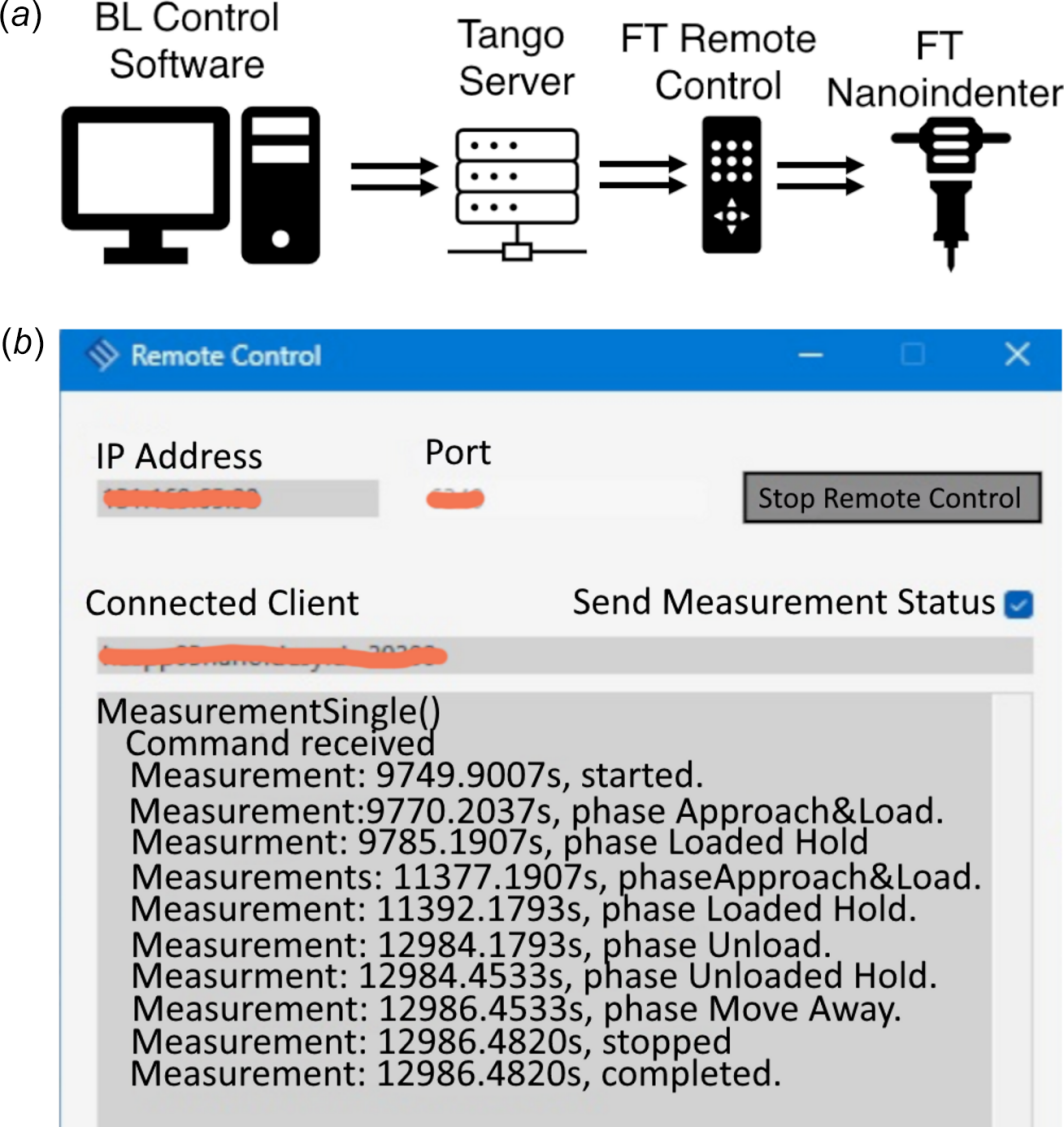
(*a*) Scheme of the communication line between the beamline control software and the nanoindenter. (*b*) Processing of commands received from the beamline control system by the nanoindenter *Remote Control*.

The communication protocol between the FemtoTools *Remote Control* software and the TANGO device server was designed to follow the typical workflow of a micromechanical test and is illustrated schematically in Fig. 2 ▸. The row labeled Communication lists the commands recognized by both the nanoindenter control software and the corresponding device server. These commands, shown in the Nanoindenter row, reflect the standard sequence of operations in a micromechanical test. At the beginning of an experiment, the user defines the mechanical testing parameters (*e.g.* strain rate, holding force, holding time, number of steps) together with the X-ray scanning parameters (scan range and dimensions, step size, and exposure time). After parameter configuration, the user initiates the find contact procedure. Once contact is established, the nanoindenter control software enters a ready state, awaiting an external software trigger to start the mechanical test and the synchronized X-ray data acquisition. This trigger, indicated by the red upward arrow in Fig. 2 ▸, initiates the entire macro-controlled process. During the test, the nanoindenter software continuously transmits status updates to the beamline device server (blue arrows), enabling real-time coordination between the mechanical actuation and X-ray data collection. While the initial contact must currently be performed manually to ensure precise control and prevent potential device damage, subsequent unloading and re-approach operations can be incorporated into predefined macros. This allows the scripted execution of cyclic loading sequences, such as initial contact → trigger → load steps → subsequent load steps, thereby enabling reproducible multi-step and cyclic *in situ* experiments. Work is ongoing to automate the initial contact procedure in a safe and reliable manner, though this is non-trivial due to the required cross-checks and safety measures. This workflow was implemented using the FemtoTools *Remote Control* software interfaced with a custom-developed TANGO device server integrated into the beamline control system. The experimental control architecture consists of three hierarchical layers [Fig. 3 ▸(*a*)]. The top layer is the user interface, provided by the Spock command-line environment of Sardana (Coutinho *et al.*, 2011 ▸). Sardana communicates with the intermediate layer composed of TANGO servers, which form the backbone of the distributed control infrastructure. TANGO is an open-source, device-oriented toolkit for managing hardware and software components in SCADA (Supervisory Control and Data Acquisition) systems (Chaize *et al.*, 2001 ▸). Sardana extends TANGO by offering a platform-independent framework with multi-language support and tools for graphical interface development.

The FemtoTools nanoindenter is connected to this environment via the custom TANGO device server, which acts as a remote TCP/IP client to the FemtoTools *Remote Control* software. Both components exchange a defined set of commands and status messages, ensuring reliable two-way communication. This setup allows the TANGO device server to relay instructions from Sardana (or any other TANGO client) to the nanoindenter, fully integrating the instrument into the automated beamline workflow. The device server was implemented in C++ and deployed on a Debian 12 Linux system. In this configuration, only the command interface of the TANGO framework was utilized, as attributes and properties were not required for the intended functionality.

Once a communication channel is established, the socket client can transmit selected commands as ASCII strings, each up to 1024 bytes in length and terminated with a carriage return and line feed (CRLF). Since the socket client continuously listens to the server, asynchronous messages and replies can be sent by the Femto *Remote Control* software at any time. The Tango server forwards this information to the scan script layer, as the indenter’s status is critical for scan-time decision-making—such as pausing a scan or waiting for a specific state before proceeding to the next step. With a predefined set of commands and messages, Sardana has all the necessary tools to fully control and monitor the nanoindenter, enabling seamless integration into the overall experiment control system as shown schematically in Fig. 3 ▸(*a*).

The developed TANGO device server was intentionally designed to ensure portability and straightforward deployment at other synchrotron facilities. It follows the standard TANGO device architecture and communicates with the nanoindenter exclusively via a TCP/IP-based ASCII command protocol provided by the FemtoTools *Remote Control* software, rendering the implementation independent of beamline-specific hardware components and custom low-level drivers. As a hardware- and application-agnostic framework, Tango Controls provides a standardized abstraction layer that enables collaborative software development across facilities and beamlines, independent of the underlying infrastructure. Consequently, the same TANGO server can be deployed with different hardware configurations and higher-level applications to perform identical tasks. Integration into another beamline environment based on TANGO and Sardana therefore requires only moderate adaptation, primarily involving device naming conventions, incorporation into existing scan macros, and network configuration. Since the nanoindenter is embedded as a native TANGO device, it can be accessed by any compatible TANGO client application. Moreover, TANGO servers are typically distributed through public repositories and can be deployed within any compliant TANGO environment. The widespread adoption of TANGO as a standard control framework at major large-scale research facilities, including DESY and ESRF, further supports software portability, long-term maintainability, and broader dissemination of the presented synchronized *in situ* nanomechanical X-ray diffraction approach.

Fig. 3 ▸(*b*) displays a screenshot of the communication interface between the FemtoTools *Remote Control* software and the TANGO device server. The interface not only shows the commands being sent to the nanoindenter but also provides real-time feedback on the execution status and corresponding device responses. Key process attributes are clearly listed in the interface, enabling the user to track the full history of commands and communication flow throughout the experiment. This level of transparency is essential for ensuring robust and synchronized operation between the mechanical testing system and beamline instrumentation. Furthermore, such a feedback system is critically important for maintaining complete control over the experiment, facilitating troubleshooting, and promptly identifying and resolving potential errors or inconsistencies during *in situ* testing.

## 3. Results

**Figure 4:**
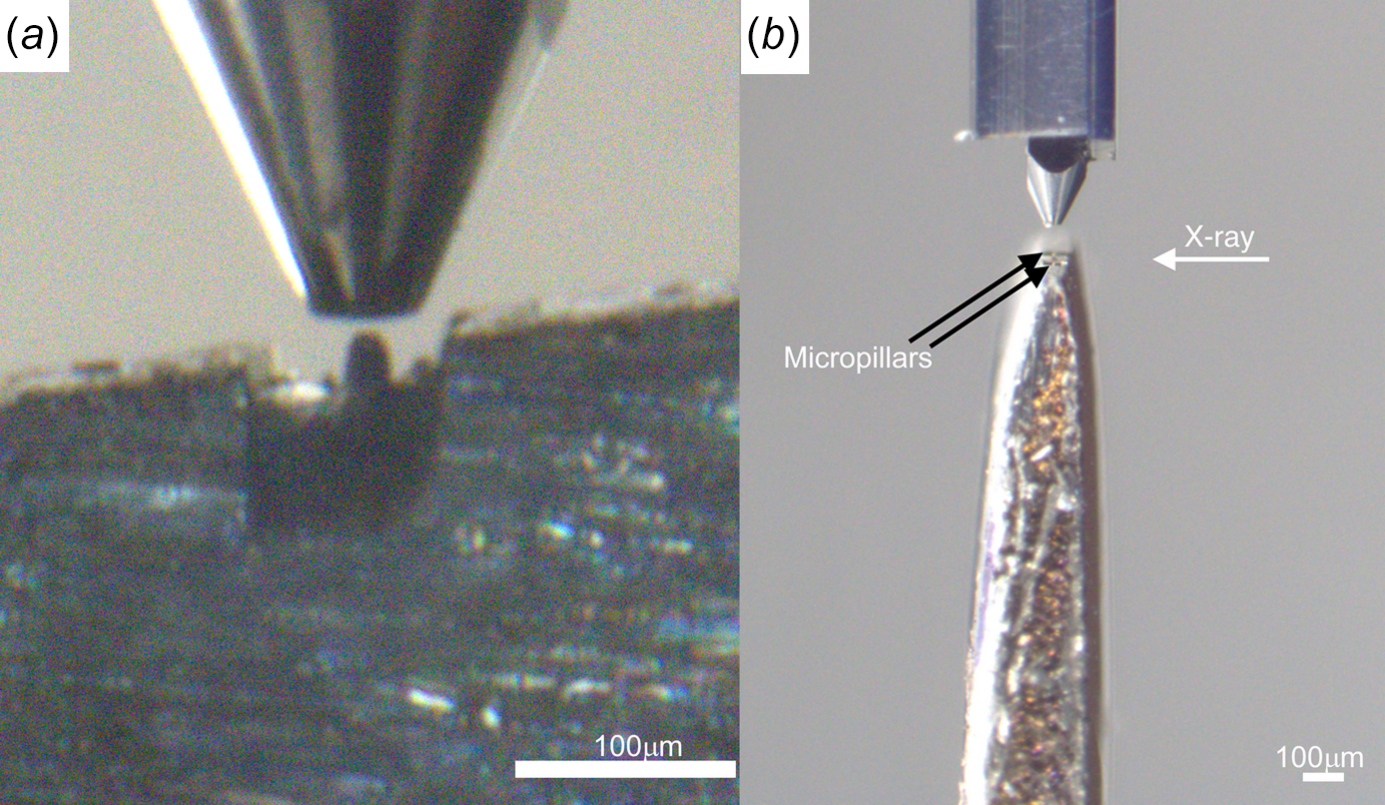
(*a*) Sample view from the front micropscope during alignments before deformation. (*b*) Sample view from the side microscope showing sample position according to the tip.

For demonstration purposes, a microcompression test was performed on an α-Ti micropillar using the FemtoTools FT-NMT04 nanoindenter equipped with a 50 µm-diameter diamond flat punch. Accurate tip–sample alignment was achieved using two optical microscopes providing orthogonal front and side views (Fig. 4 ▸). Fig. 4 ▸(*a*) shows a front-view optical image of the micropillar fabricated from bulk metal. The flat punch is centered above the FIB-machined pillar, aligned for testing. The 50 µm tip is positioned directly over the micropillar, with the front plate of the substrate in sharp focus. The slight defocus of the micropillar indicates its position slightly behind the focal plane, while the upper conical portion of the tip appears sharper than the flat punch surface. During station commissioning, the front-view camera was also used to mark the beam position for future sample alignments. In routine operation, it is employed first to bring the micropillar into the beam position, before refining the placement with the side view. Further alignment requires the side-view camera, which allows precise adjustment along the X-ray beam direction. Fig. 4 ▸(*b*) presents a side-view image, showing the tip and two FIB-fabricated areas with micropillars. From this perspective, the exact spatial relationship between the tip and micropillar can be confirmed. This view underscores that both the front and side cameras are critical for ensuring accurate alignment along the beam path, highlighting the necessity of the side-view perspective.

**Figure 5:**
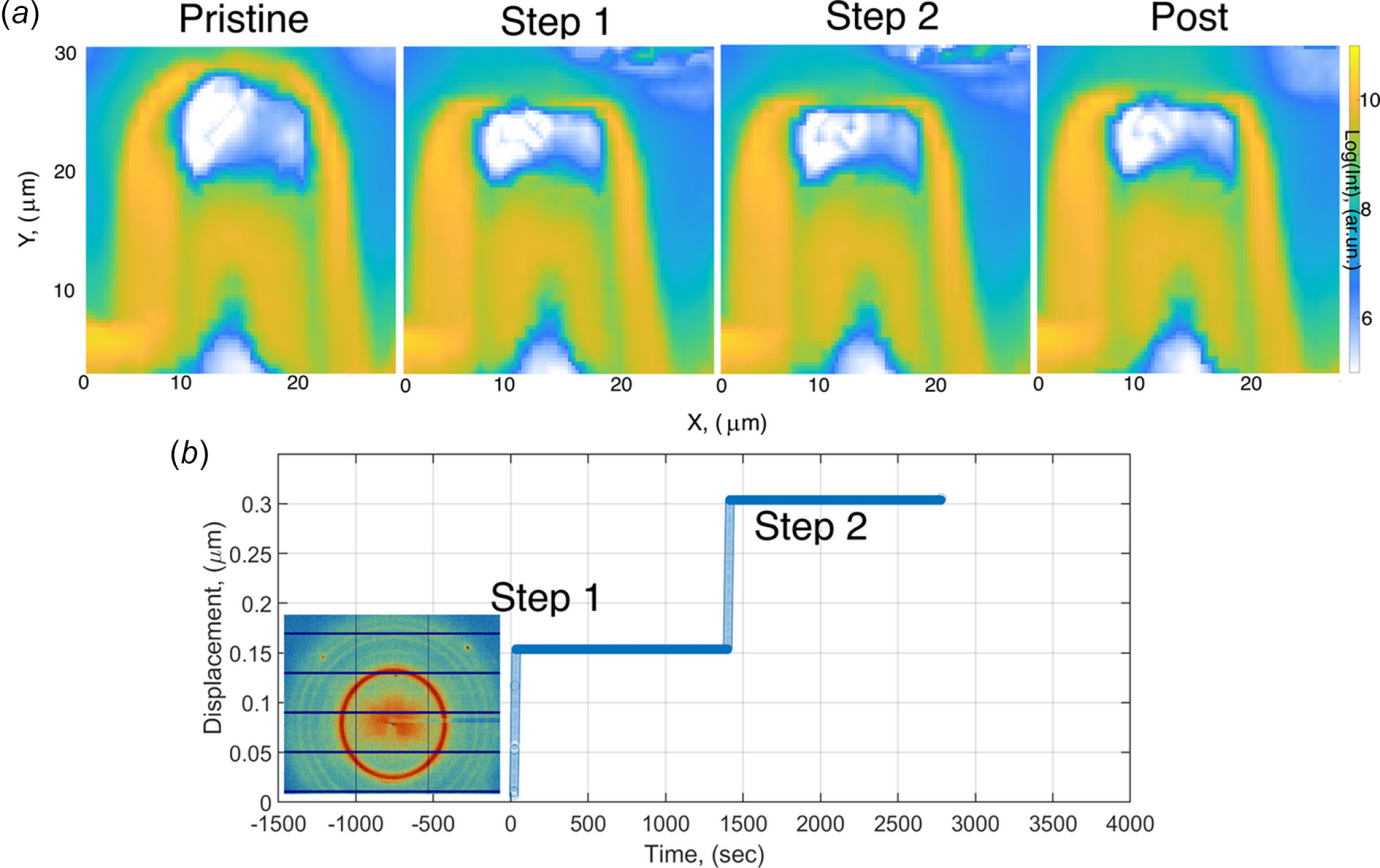
(*a*) XRF maps recorded from the Ti micropillar at different deformation stages: pristine, step1, step2 and post-deformation. (*b*) Displacement–time graph applied during uniaxial compression test. Raster scans were performed in step-scan mode over 30 µm × 35 µm with 30 × 35 points (1 µm step size, 1 s exposure per point; total scan time 1592 s including overhead; 10 s stabilization before and after each load step).

Final alignment of the micropillar with the X-ray beam was achieved using X-ray fluorescence (XRF) mapping of the titanium signal [Fig. 5 ▸(*a*)], allowing precise localization of the pillar within the laboratory coordinate system. XRF maps were obtained through raster scans under a fixed load and repeated after the compression experiment. The XRF intensity maps were constructed from the integrated counts of the Ti *K*α-line region of interest extracted from the recorded fluorescence spectra at each scan position. Fig. 5 ▸(*a*) presents a sequence of Ti XRF maps acquired at four key stages of the *in situ* uniaxial compression: pristine, during step 1, during step 2, and post-deformation. The maps, displayed on a logarithmic intensity scale, reveal the spatial distribution of the Ti fluorescence signal across the micropillar cross-section. The central region of the pristine map exhibits a saturated (white) area caused by detector overexposure. This saturation resulted from the combination of a higher local Ti concentration and a change in beam size from a submicrometre beam (for which the Amptek detector was calibrated) to a 1 µm beam. The larger beam increased the detected intensity by roughly an order of magnitude, exceeding the detector’s dynamic range and producing a no-count region. Despite this localized saturation, the overall micropillar geometry and expected dimensions are clearly discernible. Notably, the top surface, which appears rounded in the pristine scan, becomes distinctly lowered and flattened in subsequent maps, reflecting the morphological changes induced by compression. While the initial map exhibits slight lateral misalignment, indicating that the micropillar is not centered, the full pillar area remains clearly visible, particularly from the top section. The fluorescence signal from titanium remains stable throughout all stages, suggesting consistent illumination and detection of the micropillar during the experiment. Minor variations in the intensity distribution may reflect microstructural changes or surface deformation induced by the applied mechanical loading. Additionally, the XRF data allow detection of the Ga-ion distribution originating from FIB milling. Although not the focus here, tracking its redistribution under applied deformation is relevant for micromechanical studies and is therefore the subject of a separate publication (Davydok *et al.*, 2026 ▸). This set of XRF maps thus serves a dual purpose: it verifies precise beam alignment with the sample throughout the experiment and provides spatially resolved insight into the morphological changes induced by mechanical loading.

Fig. 5 ▸(*b*) displays the displacement–time graph recorded during the *in situ* compression test, corresponding to the loading steps described in Fig. 5 ▸(*a*). The experiment was performed in displacement-controlled mode at a constant strain rate of 0.1 s^−1^. The target displacement was set to 0.3 µm, equivalent to approximately 1% of the micropillar height, based on prior observations that larger displacements can cause complete failure of such brittle pillars. Two intermediate hold segments were incorporated into the loading sequence: the first at 0.15 µm (step 1) and the second at 0.3 µm (step 2) displacement. Each hold lasted 1592 s, corresponding to the time required for a complete XRD/XRF raster scan of the pristine micropillar. The XRF/XRD raster scans were performed in step-scan mode over an area of 30 µm × 35 µm (horizontal × vertical), sampled with 30 × 35 measurement points, resulting in a step size of 1 µm in both directions. The exposure time per point was set to 1 s. A representative two-dimensional diffraction pattern recorded during the raster scan is shown as an inset in Fig. 5 ▸(*b*). The diffraction signal exhibits a continuous Debye–Scherrer ring together with localized high-intensity spots. This mixed appearance reflects the presence of differently oriented crystalline regions within the illuminated volume of the micropillar, leading to a combination of ring-like scattering and discrete spot contributions. The total scan time of 1592 s includes the pure acquisition time as well as the intrinsic system overhead associated with motor movement and data handling. In addition, a stabilization buffer of 10 s was implemented both before and after each deformation step to ensure mechanical equilibration and reliable synchronization between the nanoindenter and X-ray data acquisition. These parameters define the achievable spatial resolution and temporal scale of the experiment and illustrate the throughput capabilities of the integrated system. Following the second hold, the indenter tip was retracted by 10 µm above the micropillar to conclude the measurement sequence. During both hold periods, the displacement data remained stable throughout the XRD acquisitions, indicating that the overall shape of the micropillar remained standing under load. However, XRF mapping [Fig. 5 ▸(*a*)] reveals deformation localized to the top surface, becoming flatter after step 1, a reduction in pillar height by approximately 5 µm, despite the much smaller displacement applied in the deformation test.

**Figure 6:**
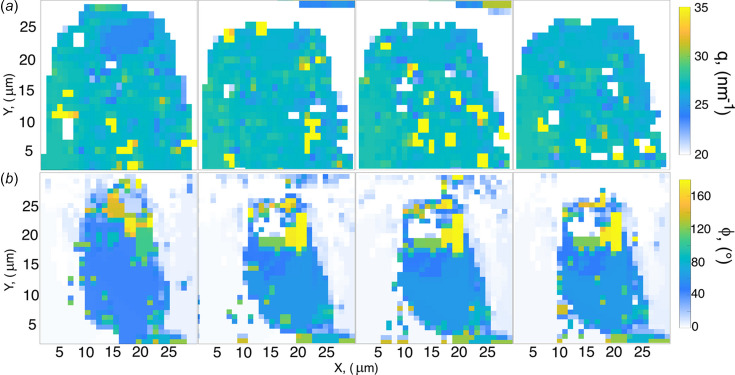
(*a*) *q*-value distribution (in nm^−1^) maps evaluated from azimuthal integration of the two-dimensional XRD data collected from the α-Ti micropillar at different deformation stages: pristine, step1, step2, and post-deformation; the displayed *q*-range (20–35 nm^−1^) represents the azimuthal spread of diffraction intensity in reciprocal space and reflects local variations in lattice spacing and microstrain. (*b*) Orientation spread (in degrees) maps obtained from radial integration of the same XRD datasets; the orientation map shows the angular intensity distribution within the range 0–180°, covering the full orientation spread of the diffraction signal.

Further analysis of the XRD patterns was performed by integrating the diffraction data into two directions, radial and azimuthal, and selecting the strongest reflection as a structural representative of the locally illuminated volume (Davydok *et al.*, 2026 ▸). Fig. 6 ▸ presents spatially resolved maps of reciprocal space coordinates (*q*-space in nm^−1^) and azimuthal angle (in degrees) distributions acquired during the *in situ* compression test. The data are arranged in four columns, corresponding to distinct deformation stages: the pristine state, step 1 of deformation, step 2 of deformation, and the post-deformed state. In Fig. 6 ▸(*a*), the maps show the distribution of reciprocal space coordinates across the micropillar surface. These values reflect local variations in lattice spacing and elastic strain within the probed volume. In pristine condition, the distribution is relatively homogeneous, *q* = 26.8 nm^−1^, and corresponds to a α-Ti (002) reflection, with only minor fluctuations in *q*-space, consistent with the initial defect-free microstructure of the micropillar. One notable exception is a region in the upper part of the micropillar, shown in light blue, with a value of *q* = 24.55 nm^−1^ α-Ti (100) (Musi *et al.*, 2024 ▸), which is in good agreement with the regions of high Ti density observed in the XRF map. As deformation progresses through step 1 and step 2, localized heterogeneities become increasingly pronounced. Regions of higher intensity (yellow areas) indicate zones of enhanced lattice distortion, likely associated with the accumulation of plastic strain and the activation of slip systems. Even after unloading, these heterogeneities persist, demonstrating that a portion of the deformation is irreversible and associated with permanent lattice reorientation and defect generation. The α-Ti (100) grain in the top part of the micropillar is no longer observed after deformation. This disappearance can be explained by the redistribution of local pre-existing strain gradients, such that the initial heterogeneity is absorbed into the overall deformation field, or by lattice rotation under applied stress leading to local reorientation. Fig. 6 ▸(*b*) shows the corresponding maps of the radial angle distribution (φ), which quantify the orientation spread of diffracted intensity in reciprocal space and thus provide insight into lattice rotation and grain reorientation during compression. In the pristine state, the distribution is relatively uniform, with φ values confined to a narrow range. With the onset of deformation (step 1 and step 2), substantial spatial variations in φ emerge, particularly in the upper regions of the micropillar. These changes reflect the initiation of crystal lattice rotations under compressive stress, consistent with dislocation activity and slip. By the post-deformation stage, significant orientation anisotropy remains, with some regions exhibiting large deviations in φ. This indicates that the microstructure has undergone permanent orientation changes that have not recovered upon unloading.

## 4. Conclusions

In this work, we have demonstrated the successful integration of a nanoindenter into the control system of a synchrotron beamline, enabling fully synchronized and automated *in situ* mechanical testing with X-ray nanodiffraction. The software-level coupling of mechanical loading and diffraction measurements provides a robust platform for high-throughput and real-time analysis of structural evolution during deformation. The capabilities of this system were validated through uniaxial compression experiments on α-Ti micropillars, where simultaneous acquisition of mechanical data and diffraction patterns allowed direct correlation between mechanical response and microstructural changes. Raster scans performed at four deformation stages (pristine, step1, step2, post-deformation) revealed the progressive development of strain heterogeneities, lattice rotations, and permanent defect structures. Overall, the results demonstrate that the integrated system not only simplifies experimental workflows but also significantly expands the scope of *in situ* synchrotron studies by providing reliable, synchronized, and spatially resolved data. This methodology opens new opportunities for systematic investigations of deformation mechanisms in small-scale materials and can be readily adapted for a broad range of materials.

## Data Availability

Data will be made available on request.
